# Recombinant TSG-6 protein inhibits the growth of capsule fibroblasts in frozen shoulder via suppressing the TGF-β/Smad2 signal pathway

**DOI:** 10.1186/s13018-021-02705-x

**Published:** 2021-09-15

**Authors:** Zhongfan Liu, Yongrong Pei, Hao Zeng, Yibo Yang, Meng Que, Yuhui Xiao, Jing Wang, Xiaojun Weng

**Affiliations:** Department of Joint and Sports Medicine, Hunan Provincial People’s Hospital (The First Affiliated Hospital of Hunan Normal University), No. 61, West Jiefang Road, Furong District, Changsha, Hunan 410005 People’s Republic of China

**Keywords:** Recombinant TSG-6 protein, TGF-β/Smad2 signaling pathway, Frozen shoulder capsule fibroblasts, Cell proliferation, Apoptosis, Inflammatory response

## Abstract

**Background:**

The tumor necrosis factor-stimulated gene-6 (TSG-6) has been confirmed to inhibit inflammation. It is now generally accepted that local inflammatory stimulation around shoulder capsule causes proliferative fibrosis. This study aims to investigate the mechanism of recombinant TSG-6 protein inhibiting the growth of capsule fibroblasts in frozen shoulder via the TGF-β/Smad2 signal pathway.

**Methods:**

Human frozen shoulder capsule tissue was taken for primary and passage culture, and the 3rd generation fibroblasts from pathological frozen shoulder capsule were treated with different concentrations of recombinant TSG-6 protein, or with TGF-β1 agonist SRI-011381. Immunoconfocal analysis was used to identify the isolated fibroblasts, and MTT assay, colony formation assay, and flow cytometry were used to detect the viability, proliferation, and apoptosis rate of fibroblast. The contents of fibrosis and inflammation indexes COL1A1, TNF-α, IL-6, and IL-1β in the cell supernatant were detected using ELISA and then further examined by qRT-PCR. The expression of Bax, Bcl-2, and proteins related to TGF-β/Smad2 pathway were detected by Western Blot.

**Results:**

Compared with the blank control group, fibroblasts intervened with TSG-6 (2 μg and 5 μg) showed significantly decreased viability and proliferation ability and enhanced cell apoptosis, concurrent with the reductions in Bcl-2 expression; COL1A1, TNF-α, IL-6, and IL-1β levels; and the expression of TGF-β1 and phosphorylated Smad22, and an increase in Bax expression, while SRI-011381 treatment would reverse the effect of recombinant TSG-6 protein.

**Conclusion:**

Recombinant TSG-6 protein inhibited the growth of primary fibroblasts from human frozen shoulder capsule by suppressing the TGF-β/Smad2 signaling pathway.

## Introduction

Frozen shoulder, also known as scapulohumeral periarthritis, is a common shoulder disease characterized by progressive spontaneous pain and limited range of motion of the glenohumeral joint, consequently leading to difficulty with daily activities and significant disability [1]. Factors that may contribute to frozen shoulder include shoulder surgery, trauma, heart disease, prolonged immobilization, diabetes, thyroid dysfunction, genetic factors, autoimmune disease, and hyperlipidemia [2]. A synovial hyperplasia with increased vascularity that occurs in the early stages results in fibrosis in the subsynovium and synovium of capsular tissue, a condition that begins as an immune response followed by inflammatory synovitis and capsule fibrosis [3]. Recent research has demonstrated that fibroblasts have an activated phenotype in frozen shoulder, which is associated with dysregulation of inflammatory cytokines [4].

Tumor necrosis factor (TNF)-α–stimulated gene/protein 6 (TSG-6), as a multifunctional endogenous protein, is a pivotal natural regulator of inflammation, and it has been demonstrated to mediate pro-inflammatory cytokine cascades and strengthen the repair of injured tissues in several animal models [5]. Recent research has suggested that TSG-6 and hepatocyte growth factor restrict the progression of fibrosis by suppressing the activation of myofibroblasts and smooth muscle cells [6]. Moreover, TSG-6 has been reported to have anti-inflammatory and chondroprotective effects in a mouse model of inflammatory arthritis [7]. In response to stimulation of pro-inflammatory cytokine TNF-α or interleukin (IL)-1, TSG-6 is secreted by a variety of cells, such as fibroblasts [8]. However, the role and mechanism of TSG-6 protein in regulating the growth of capsule fibroblasts in frozen shoulder require further exploration.

Martin et al. have showed that interactions of TSG-6 with the inter-α-inhibitor heavy chain 5 promote tumor growth factor β1 (TGFβ1)-dependent fibroblast to myofibroblast differentiation [9]. The TGF-β superfamily is composed of highly variable molecules, which include bone morphogenetic proteins, growth differentiation factors, glial neurotrophic factors, activins, and inhibins, and it plays multiple biological roles in kidney inflammation, proliferation, fibrosis, and apoptosis [10]. TGF-β, as a critical compliant multifunctional cytokine, is involved in numerous human diseases, exerting its biological functions through TGF-β/Smad2-mediated and non-Smad2-mediated signaling pathways [11]. Furthermore, previous studies have reported that TGF-β/Smad2 is a primary pathway in the progressive fibrosis processes [12] and elevated level of TGF-β is commonly related to fibrosis and disease progression [13]. In addition, besides regulation on tissue fibrosis, TGF-β1 also involved in many biological responses, such as cell proliferation, differentiation, autophagy, apoptosis and immune response [14]. Importantly, Xue et al. have revealed that Smad4 silencing inhibits chronic inflammation and fibrosis in joint tissues by suppressing the TGF-β/Smad pathway [15].

Although extensive research has been carried out on frozen shoulder, no single study explores the mechanism of recombinant TSG-6 protein regulation on frozen shoulder. In present study, therefore, it is speculated that recombinant TSG-6 protein may exert an effect on inhibiting the growth of capsule fibroblasts in frozen shoulder via suppressing the TGF-β/Smad2 signaling pathway. The results in the present study may provide a new mechanism for the treatment of frozen shoulder in the future.

## Materials and methods

### Sample collection

Diseased shoulder capsule samples were obtained from 5 patients diagnosed with frozen shoulder in the Department of Joint and Sports Medicine, Hunan Provincial People’s Hospital. The capsule tissues disused during operation were collected from those who had signed informed consent and had not undergone shoulder joint cavity perfusion or other medication within 3 months before surgery. The experiment was approved by the ethics committee of Hunan Provincial People’s Hospital.

### Primary fibroblast cell acquisition and culture

The hyperplastic tissue of the shoulder bursa was taken under arthroscopy, and then washed three times with D-hanks solution (containing 100 μg/ml streptomycin). The adipose tissue and small blood vessels on the capsule tissues were scraped off, and the tissues were cut into 0.5-mm^3^ pieces. The clean tissue pieces were evenly placed on the wall of the culture flask with a pipette, and placed in an incubator (5% CO_2_, 37°C, saturated humidity) for 2–3 h with the tissue pieces facing upward. After the tissue pieces were completely adhered to the wall, the culture flask was slightly inverted, and DMEM containing 20% fetal bovine serum (2 mL) was added to fully infiltrate the tissue pieces. The pieces were then placed in a CO_2_ incubator, and the cell culture medium was replaced every 2–3 days with the supplemented cell culture medium slightly above the bottom of the culture flask. On the days 12–15, cells were sprouted around the tissue pieces, and when the sprouted cells were connected, they were passaged at 1:2 ratio. The cells of the 3rd generation were used for following experiments.

### Immunoconfocal analysis

Immunoconfocal analysis was used to identify the isolated fibroblasts. Cells were plated in glass-bottom culture dishes at a density of 1.5 × 10^5^ per well. After 24 h of incubation, cells were fixed with 4% paraformaldehyde (Solarbio, P1110) for 15 min at room temperature and then washed 3 times with PBS, and 0.3% Triton X-100 (Sigma, Triton™ X-100) was added for incubation for 15 min at room temperature. Afterwards, the cells were washed 3 times with PBS, incubated with 5% bovine serum albumin at room temperature for 2 h. Cells were incubated with primary antibody against Vimentin (CST, 5741S, 1:500) overnight at 4°C. On the next day, the cells were washed 3 times with PBS followed by incubation with goat anti-Rabbit fluorescein secondary antibody (ThermoFisher, A-11034, 1:1000) for 1 h at room temperature avoiding light exposure. DAPI was incubated with the cells at a concentration of 5 mg/L (Sigma, D8417) for nuclear staining away from light at room temperature for 5 min. Photographs were taken using a Leica DMI6000 confocal microscope with 63 × oil immersion lens and processed by LAS AF software.

### Experimental grouping

The 3rd generation fibroblasts from frozen shoulder capsule were made into 1 × 10^5^/mL cell suspension (6 ml) and then divided into three aliquots and cultured in culture flasks separately. After 24 h of incubation, the cells were treated with 0, 2, or 5 μg of recombinant TSG-6 protein, and accordingly divided into the control group, 2 μg TSG-6 group, and 5 μg TSG-6 group. After 4 h, hydrochloride SRI-011381 (10 μM) was added into the culture medium. Forty-eight hours later, the cells were subjected to subsequent detection.

### MTT assay

Three groups of treated cells were seeded in 96-well plates at a concentration of 1 × 10^4^ cells/ml. The 96-well plates were moved into the incubator and cells were incubated for a certain time (24, 48, 72 h). After the incubation, the supernatant was discarded, and 5 mg/ml MTT solution and PBS100 were added into each well (avoiding air bubbles). After 4–6 h of incubation, the cell culture was terminated and the culture solution was aspirated off. An equal amount of DMSO was added into each well, followed by shaking for 30 s. The absorbance values of each well at 570 nm were measured by a microplate reader.

### Colony formation assay

Three groups of treated cells were inoculated in 6-well plates at a density of 30,000 cells/mL and cultured in fresh medium. Colony formation assay was implemented according to the kit instructions (Invitrogen, CA, USA). Crystalline violet-stained clones of > 50 cells were observed under a microscope.

### Flow cytometry

The cells were made into single cell suspension, collected after centrifugation at 2000 r/min, washed twice in PBS, and resuspended in binding buffer. Annexin-V-FITC (5 μl) and PI staining solution (5 μl) were added to 195 μL of cell suspension containing approximately 10^5^ cells and then incubated for 10 min without light exposure before detection on apoptosis rate using a flow cytometer (BD Biosciences, Suzhou, China).

### Enzyme-linked immunosorbent assay (ELISA)

The levels of TNF-α, IL-6, IL-1β, and COL1A1 were quantified using ELISA kits (Jianglai, Shanghai, China). Absorbance was measured at 450 nm using a microplate reader (S/N 415-2687, Omega Bio-Tek, Ortenberg, Germany).

### Quantitative real-time polymerase chain reaction (qRT-PCR)

Total RNA was extracted from each group of cells using TRIzol and was subjected to reverse transcription into cDNA using a reverse transcription kit. Primers for qRT- PCR were synthesized by Sangon Biotech (Shanghai) Co., Ltd., according to the sequences shown in Table 1. Then, 10 μL cDNA was taken and PCR reactions were performed on a Roche 480 real-time fluorescence PCR instrument using SYBR® Green RealTime PCR Master Mix. GAPDH was used as an internal reference using the 2^−∆∆Ct^ method [16].

**Table 1:** Primer information

Name	Sequences
COL1A1-F	CTGCTGGACGTCCTGGTGAA
COL1A1-R	ACGCTGTCCAGCAATACCTTGAG
TNF-α-F	TAAGCAACAAGACCACCACTTC
TNF-α-R	TCTCCAGATTCCAGATGTCAGG
IL-6-F	ACTCACCTCTTCAGAACGAATTG
IL-6-R	CCATCTTTGGAAGGTTCAGGTTG
IL-1β-F	AAACAGATGAAGTGCTCCTTCCAGG
IL-1β-R	TGGAGAACACCACTTGTTGCTCCA
GAPDH-F	CGGAGTCAACGGATTTGGTCGTA
GAPDH-R	AGCCTTCTCCATGGTGGTGAAGAC

*Note*: *F* forward primer, *R* reverse primer

### Western blot

The total protein was extracted from the cells by adding RIPA lysis buffer containing protease inhibitor. The protein concentration was measured by BCA kits (Beyotime Biotechnology Co., Ltd., Shanghai, China). Appropriate amount of 5 × SDS⁃PAGE loading buffer was mixed with proteins (15 μL per well) for 10% SDS-PAGE electrophoresis at 300 mA. The proteins were wet transferred to PVDF membrane and then sealed in 5% skim milk powder solution at room temperature for 1 h. The PVDF membranes were incubated with the primary antibodies against Bax (ab32503, 1:1000, Abcam, Cambridge, MA, USA), Bcl-2 (ab32124,1:1000, Abcam, Cambridge, MA, USA), TGF-β1 (ab215715, 1:1000, Abcam, Cambridge, MA, USA), p-Smad2 (ab280888, 1:1,000, Abcam, MA, USA), Smad2 (ab40855, 1:1,000, Abcam, MA, USA), and GAPDH antibody (#5174, 1:1,000, Cell Signaling Technology, Beverly, MA, USA) at room temperature on a shaking table overnight. The membranes were washed with TBST and then incubated at room temperature for 1 h with secondary antibody (ab6728, 1:2000, Abcam, Cambridge, MA, USA). After TBST washing, the membranes were uniformly dropped with chemiluminescent solution, and the results were analyzed using Image J, with GAPDH as the internal reference.

### Statistical analysis

All experiments were repeated three times unless otherwise stated. Statistical analysis of data was performed using SPSS 18.0 (IBM Corp., Armonk, NY, USA) and GraphPad Prism 8.0 (GraphPad Software Inc.), and data were displayed as mean ± standard deviation (SD). T-test was used for comparison between two groups, and one-way analysis of variance test for comparison among multiple groups, with Tukey’s multiple comparison test for post hoc analysis. *P* < 0.05 was regarded as statistically significant.

## Result

### Fibroblasts were isolated successfully

**Fig. 1:**
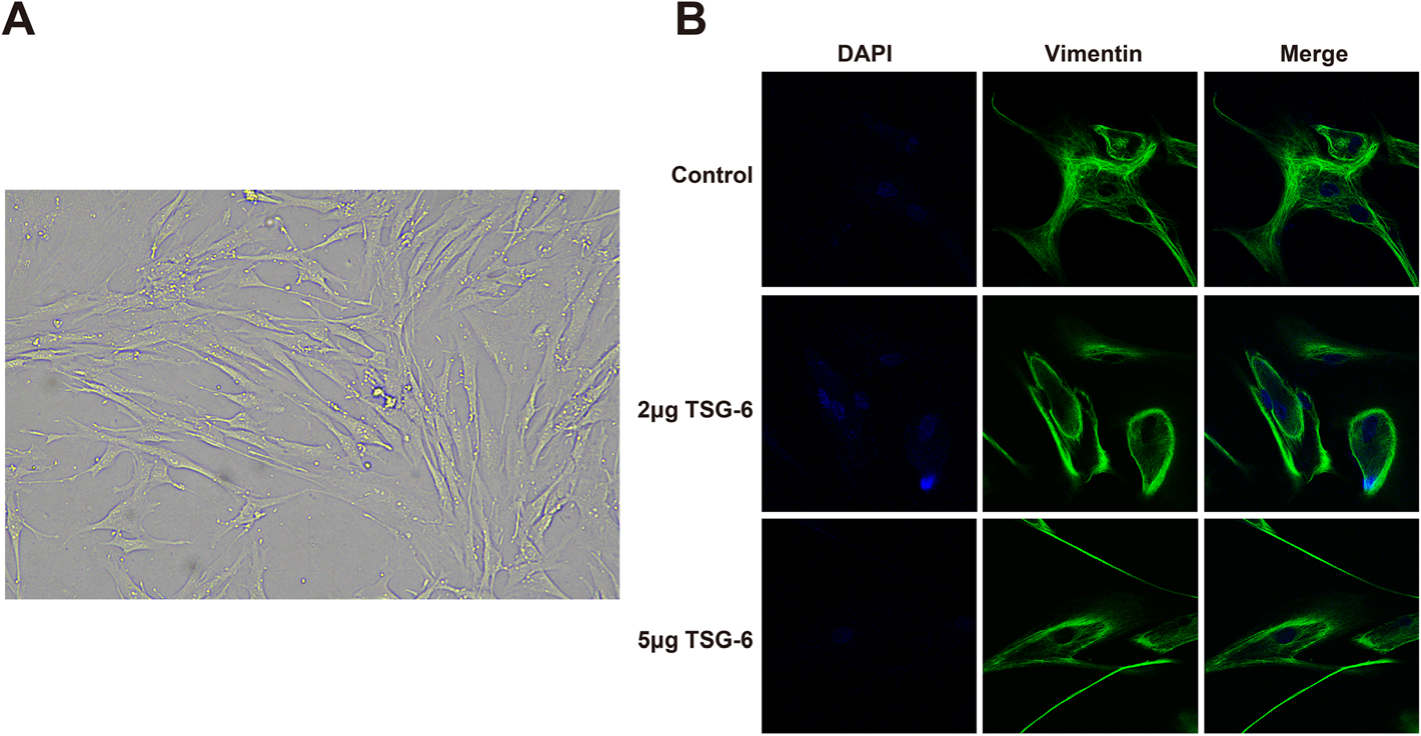
Identification of fibroblasts. Notes: **A** Diagram of primary fibroblasts. **B** The expression of Vimentin analyzed by immunoconfocal analysis

The isolated cells were observed under an inverted microscope with longer shuttle or triangular shape, which was consistent with the characteristics of fibroblasts (Fig. 1A). The positive rate of fibroblasts obtained was 98% by immunoconfocal staining analysis for vimentin (Fig. 1B). It was confirmed that the cells isolated from the bursal tissue were fibroblasts.

### Recombinant TSG-6 protein inhibits the proliferation and promotes the apoptosis of fibroblasts

**Fig. 2:**
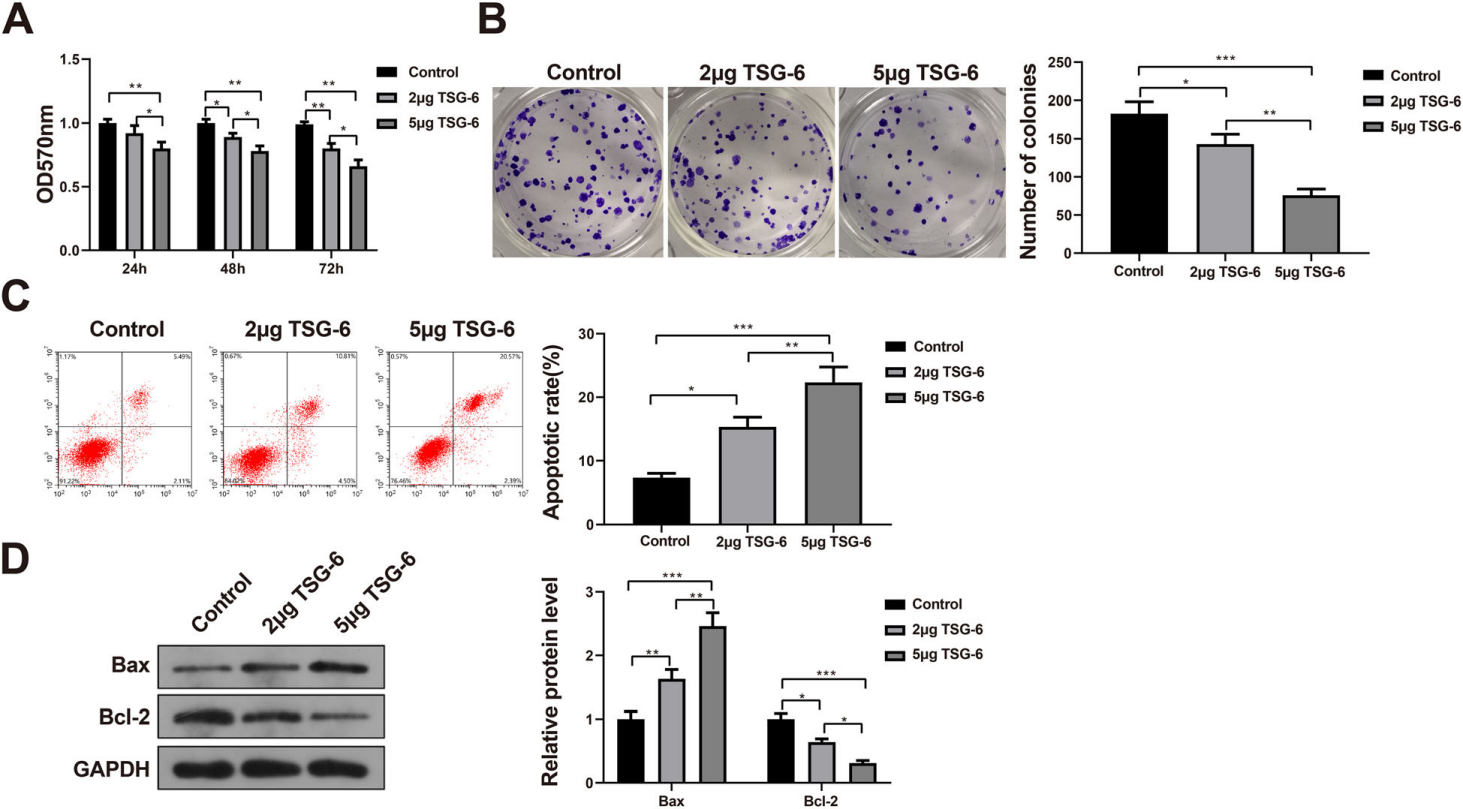
Recombinant TSG-6 protein inhibited the proliferation and promotes the apoptosis of fibroblasts. Notes: **A** MMT assay was used to detect the viability of fibroblasts in each group. **B** Colony formation assay was performed to determine the proliferation of fibroblasts in each group. **C** The apoptosis of fibroblasts in each group was detected by flow cytometry. **D** Western Blot was carried out to examine the levels of Bax and Bcl-2 of fibroblasts in each group. *P < 0.05, **P < 0.01, ***P < 0.001

To investigate the effects of recombinant TSG protein on fibroblast proliferation and apoptosis, fibroblasts in each group were cultured with recombinant TSG-6 protein for respectively 24 h, 48 h and 72 h, and cell viability of fibroblasts was determined using MTT assay. Fibroblast viability was significantly lower in the cells of the 2 μg TSG-6 group and the 5 μg TSG-6 group compared with the blank control group (*P* < 0.05). Meanwhile, the inhibitory effect on fibroblast viability was obviously higher in the cells of the 5 μg TSG-6 group compared with the 2 μg TSG-6 group (*P* < 0.01, Fig. 2A). The colony formation assay and flow cytometry were performed to examine the proliferation and apoptosis of fibroblasts in each group, and the results exhibited that the proliferation of fibroblasts was significantly lower and the apoptosis of fibroblasts was notably higher in cells of the 2 μg TSG-6 and 5 μg TSG-6 groups compared with the blank control group (*P* < 0.05). Moreover, the inhibitory effect on proliferative capacity and the promoting effect on apoptotic capacity of fibroblasts were markedly higher in cells of 5 μg TSG-6 group compared with the 2 μg TSG-6 group (*P* < 0.01, Fig. 2B, C). The levels of apoptosis-related proteins Bax and Bcl-2 were examined, and the results demonstrated that the level of Bax was clearly increased while the level of Bcl-2 was significantly decreased in the cells of 2 μg TSG-6 group and 5 μg TSG-6 group than those of the blank control group (*P* < 0.05 or *P* < 0.01). Furthermore, the level of Bax was further elevated whereas the level of Bcl-2 was further declined in the cells of 5 μg TSG-6 group compared with the 2 μg TSG-6 group (*P* < 0.05 or *P* < 0.01, Fig. 2D). The above indicated that recombinant TSG-6 protein inhibited the proliferation and promoted the apoptosis of fibroblasts.

### Recombinant TSG-6 protein inhibits fibroblast fibrosis and inflammatory response and suppressed TGF-β/Smad2 signaling pathway

**Fig. 3:**
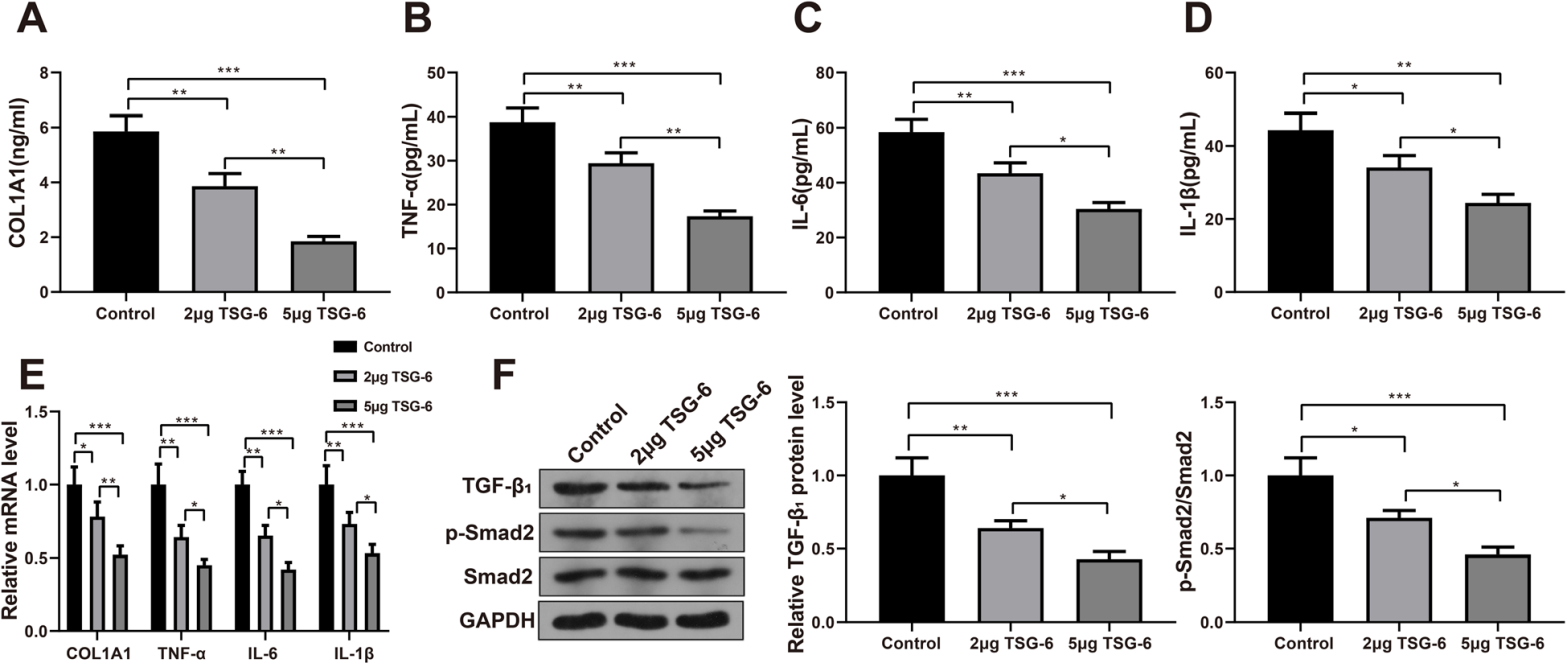
Recombinant TSG-6 protein inhibited fibroblast fibrosis and inflammatory response and suppressed TGF-β/Smad2 signaling pathway. Notes: **A**–**D** ELISA was used to detect the contents of COL1A1, TNF-α, IL-6, and IL-1β in the cell supernatant. **E** qRT-PCR was performed to determine the levels of COL1A1, TNF-α, IL-6, and IL-1β in the cells. **F** Western Blot was used to detect the level of TGF-β1 and phosphorylation level of Smad2 in cells. *P < 0.05, **P < 0.01, ***P < 0.001. ELISA, enzyme-linked immunosorbent assay

Firstly, ELISA was used to detect the contents of COL1A1, TNF-α, IL-6, and IL-1β in the cell supernatant. Compared with the blank control group, the contents of COL1A1, TNF-α, IL-6, and IL-1β in the cell supernatant of the 2 μg TSG-6 group and 5 μg TSG-6 group were significantly reduced (*P* < 0.05 or *P* < 0.01), and they were further decreased in the 5 μg TSG-6 group compared with the 2 μg TSG-6 group (*P* < 0.05 or *P* < 0.01, Fig. 3A–D). The levels of COL1A1, TNF-α, IL-6, and IL-1β in the cells were also verified by qRT-PCR (Fig. 3E).

The TGF-β/Smad2 signaling pathway plays an important role in fibrosis, and it has been documented that TSG-6 regulates the TGF-β/Smad2 signaling pathway in keloid fibroblasts. Therefore, we examined the level of TGF-β1 and phosphorylation level of Smad2 in fibroblasts using Western Blot. Compared with the blank control group, the level of TGF-β1 and the phosphorylation level of Smad2 in the 2 μg TSG-6 and 5 μg TSG-6 groups were clearly inhibited (*P* < 0.05 or *P* < 0.01), and they were further suppressed in the 5 μg TSG-6 group compared with the 2 μg TSG-6 group (*P* < 0.05, Fig. 3F). The above implied that recombinant TSG-6 protein inhibited fibroblast fibrosis and inflammation and suppressed the activation of the TGF-β/Smad2 signaling pathway.

### Recombinant TSG-6 protein restrains the growth of fibroblasts via TGF-β/Smad2 pathway

**Fig. 4:**
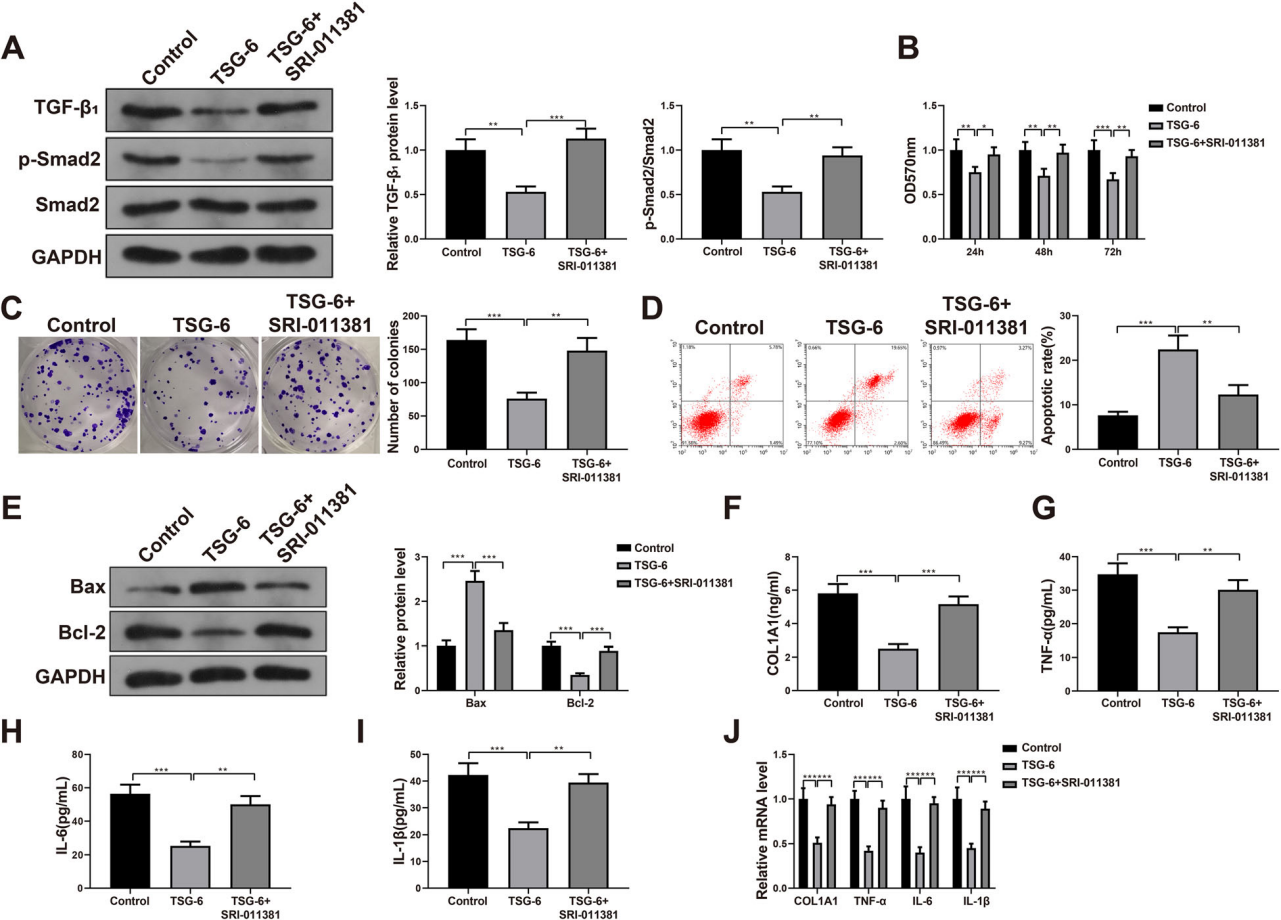
Recombinant TSG-6 protein restrained the growth of fibroblasts via the TGF-β/Smad2 pathway. Notes: **A** Western Blot was used to detect the level of TGF-β1 and phosphorylation level of Smad2 in cells. **B** MMT assay was employed to detect the viability of fibroblasts in each group. **C**, **D** Colony formation assay (**C**) and flow cytometry (**D**) were used to detect the proliferation and apoptosis of fibroblasts. **E** Western Blot was carried out to examine the levels of Bax and Bcl-2 of fibroblasts in each group. **F**–**I** ELISA was used to detect the contents of COL1A1, TNF-α, IL-6, and IL-1β in the cell supernatant. **J** qRT-PCR was performed to determine the levels of COL1A1, TNF-α, IL-6, and IL-1β in the cells. *P < 0.05, **P < 0.01, ***P < 0.001. ELISA, enzyme-linked immunosorbent assay

To verify whether the recombinant TSG-6 protein could affect capsule fibroblasts from frozen shoulder via the TGF-β/Smad2 signaling pathway, we selected recombinant TSG-6 protein (5 μg) and TGF-β1 agonist (10 μM) for the next experiment. The level of TGF-β1 and the phosphorylation level of Smad2 were remarkably enhanced in the TSG-6 + SRI-011381 group compared with the TSG-6 group (*P* < 0.05 or *P* < 0.01, Fig. 4A), which indicated that SRI-011381 successfully activated the TGF-β/Smad2 signaling pathway. Compared with the TSG-6 group, the cell viability and proliferation were obviously raised and the apoptosis was significantly reduced in the TSG-6+SRI-011381 group (*P* < 0.01, Fig. 4B–D). The results of Western Blot showed that the TSG-6 group had obviously increased Bax level and significantly decreased Bcl-2 level compared with the blank control group. While compared with the TSG-6 group, the level of Bax was markedly declined and the level of Bcl-2 was significantly raised in the TSG-6 + SRI-011381 group (*P* < 0.001, Fig. 4E). The above implied that activation of the TGF-β/Smad2 signaling pathway counteracted the effects of recombinant TSG-6 protein on fibroblast proliferation and apoptosis.

ELISA results showed that compared with the blank control group, the contents of COL1A1, TNF-α, IL-6, and IL-1β in the cell supernatant of TSG-6 group were notably decreased, whereas they were clearly elevated in the TSG-6 + SRI-011381 group than those in the TSG-6 group (*P* < 0.01 or *P* < 0.001, Fig. 4F-I). The results were further verified by qRT-PCR (Fig. 4J). The findings suggested that activation of the TGF-β/Smad2 signaling pathway counteracted the inhibition of recombinant TSG-6 protein on cell fibrosis and the inflammation.

In conclusion, recombinant TSG-6 protein mediated the TGF-β/Smad2 signaling pathway to suppress fibroblast growth.

## Discussion

Frozen shoulder is one of the most common but still challenging clinical conditions for orthopaedic surgeons [1]. Previously, a study reported that at an average follow-up of 52.3 months, 59% of 223 patients with primary frozen shoulder were near normal, 35% had mild/moderate symptoms, and 6% had severe symptoms [17]. Therefore, it is needed to find better strategies for the treatment of frozen shoulder. In order to probe the effect of recombinant TSG-6 protein in frozen shoulder, a serial of experiments were carried out in this study and the results implicated that recombinant TSG-6 protein inhibited the growth of capsule fibroblasts in frozen shoulder via suppressing the TGF-β/Smad2 signaling pathway.

One of our major findings was that recombinant TSG-6 could inhibit fibroblast viability and proliferation and promoted the apoptosis of fibroblasts. Rotator cuff tears are considered a common risk factor of shoulder pain and dysfunction, including frozen shoulder [18]. TSG-6 reportedly regulates the function of tendon derived stem cells on improving the healing of rotator cuff [19]. It could be generated within synovium and cartilage of arthritic joints [20] and could produce its anti-inflammatory effects through functioning as a pluripotent regulator of chemokines by modulating chemokine/GAS interactions [21]. Consistently with our results, the implication of TSG-6 in aseptic inflammations has also been showed in other disease, including murine models of experimental arthritis [22], rat model of osteoarthritis [23], and acute pancreatitis [24]. Watanabe et al. have manifested that TSG-6 markedly inhibited the proliferation of human aortic smooth muscle cells in a concentration-dependent manner, with a maximum reduction of 31% at 300 ng/mL [25]**.** Additionally, a recent study has analyzed that overexpression of TSG-6 suppressed cell proliferation and induced apoptosis of keloid fibroblasts [26]. Data obtained from a study reported by Wang et al. have illustrated that the TSG-6 attenuates both the expansion of fibrous matrix and inflammation in the injured liver [27]. Moreover, Zhang et al. has exhibited that subconjunctival injection of TNF-α pre-stimulated bone marrow-derived mesenchymal stem cells enhanced the anti-fibrotic and anti-inflammatory effects of ocular alkali burns, probably due to the upregulation of TSG-6 and PTGS2 expression [28]. Of note, the results of ELISA and qRT-PCR in this study exhibited that recombinant TSG-6 protein inhibited fibroblast fibrosis and inflammatory response in frozen shoulder.

This work adds to literature on the function of TSG-6 in frozen shoulder; nevertheless, the specific mechanism of how recombinant TSG-6 acts on frozen shoulder is still not fully delineated. Accumulating evidence suggests that TGF-β/Smad signaling pathway plays an essential role in fibrosis [29–31]. TGF-β/Smad3 signaling pathway is activated in synovium of patients with rheumatoid arthritis, being involved with Smad7 deficiency, and enhancement of Th17 and Th1 immune response [32]. Furthermore, Li et al. have demonstrated that TSG-6 inhibits the growth of keloid fibroblasts by triggering apoptosis in keloid fibroblasts, which may be related to the TGF-β1/Smad pathway [33]. Therefore, we detected the level of TGF-β1 and phosphorylation level of Smad2 in fibroblast and the results suggested that recombinant TSG-6 protein obviously inhibited the activation of the TGF-β/Smad2 signaling pathway. Previously, a study has demonstrated that TSG-6 suppresses the BMP-4/Smad2 signaling pathway and odontogenic/osteogenic differentiation in dental pulp stem cells [34]. To validate whether recombinant TSG-6 protein could inhibit the growth of capsule fibroblasts in frozen shoulder through the TGF-β/Smad2 signaling pathway, we selected 5 μg of recombinant TSG-6 protein and 10 μM of TGF-β1 agonist. The findings suggested that activation of the TGF-β/Smad2 signaling pathway counteracted the effects of recombinant TSG-6 protein on fibroblast proliferation, apoptosis, and inflammation, demonstrating that recombinant TSG-6 protein mediated the TGF-β/Smad2 signaling pathway to suppress fibroblast growth.

In the present study, we used several methods to explore the effect and mechanism of recombinant TSG-6 protein in frozen shoulder. The results of this study, however, have to be seen in light of some limitations. For instance, the study was conducted only at the cellular level and no experiments were performed at the animal level. In this regards, more evidence are required to validate the results of this study.

## Conclusion

In conclusion, the present study is the first to investigate the role of recombinant TSG-6 protein in inhibiting the growth of capsule fibroblasts in frozen shoulder via the TGF-β/Smad2 signaling pathway. Our results provide evidence of biological mechanisms of TSG-6 in regulating capsule fibroblasts in frozen shoulder, therefore TSG-6 may be proposed a potential target for the treatment of frozen shoulder.

## Data Availability

The datasets used or analyzed during the current study are available from the corresponding author on reasonable request.

## Abbreviations

TSG-6: Tumor necrosis factor-stimulated gene-6
TNF: Tumor necrosis factor
IL: Interleukin
TGFβ1: Tumor growth factor β1
